# Supplementary material for: Secure analysis in cognitive satellite-terrestrial networks with untrusted LEO relaying and friendly jamming
Source: PLoS One. 2025 Aug 11;20(8):e0328729. doi: 10.1371/journal.pone.0328729 (PMC12338819; doi:10.1371/journal.pone.0328729)

Secure analysis in cognitive satellite-terrestrial networks with untrusted LEO relaying and friendly jamming

Peng Zhang[1], Jin Gao[1]*,

1the School of Physics and Electronic Information, Weifang University, Weifang, Shandong, China

These authors contributed equally to this work.
* Corresponding author. E-mail address: gaojin_wfu@163.com

Abstract

This study examines physical layer security in a cognitive satellite-terrestrial network where a ground user (GU) transmits confidential data to a Geostationary Earth Orbit (GEO) satellite via an untrusted Low Earth Orbit (LEO) relay adopting the Amplify-and-Forward (AF) relay strategy. To counter eavesdropping risks from the relaying LEO satellite, a friendly LEO jammer emits artificial noise. The secrecy rate is analyzed under cognitive radio constraints, considering interference thresholds, relay gain, and GU-LEO link shadowing. Numerical results show that increasing the interference threshold initially improves the secrecy rate, but saturates when the maximum transmit power constraint at the GU is reached. When this maximum power constraint is removed, the interference threshold can be adjusted to optimize the secrecy rate. Additionally, we observe that lighter shadowing conditions generally enhance the secrecy rate, even though they improve the untrusted relay's channel. These findings offer valuable insights into optimizing security in cognitive satellite-terrestrial networks.

Introduction

The integration of satellite and terrestrial networks has become an essential component of modern communication systems, particularly as the demand for seamless, high-speed global coverage continues to grow [1–6]. Low Earth Orbit (LEO) satellites, due to their proximity to the Earth, provide low-latency and high-bandwidth capabilities, positioning them as ideal relay candidates within hybrid satellite-terrestrial networks [7]. These LEO satellites not only enhance connectivity between ground users (GUs) and Geostationary Earth Orbit (GEO) satellites, facilitating efficient long-distance communications [8], but they also play a crucial role in remote sensing applications [9]. By acting as relay nodes, LEO satellites enable the rapid transmission of high-resolution imagery and real-time data from Earth observation systems, thus supporting critical functions such as environmental monitoring, disaster response, and resource management. As such, the collaboration between LEO satellites and terrestrial infrastructures is vital for advancing both communication and remote sensing capabilities, ultimately contributing to informed decision-making and improved global connectivity [10, 11].

However, with the increasing reliance on LEO satellites as relays, security concerns become even more pronounced when these relay satellites are considered untrusted. An untrusted LEO satellite may not only forward the GU's signal to the GEO satellite but

may also attempt to eavesdrop on the transmitted information, posing a significant threat to the confidentiality of the communication. The challenge is further complicated by the fact that the untrusted LEO relay may have access to the codebook used by the GU, enabling it to decode the relayed messages and potentially manipulate or disrupt the data flow. Moreover, any physical layer technique that aims to improve the connection between the GU and the GEO satellite, such as increasing the transmit power or optimizing signal transmission, will simultaneously enhance the connection between the GU and the LEO relay. This dual enhancement complicates efforts to secure communication, as strategies intended to strengthen the legitimate channel may simultaneously amplify the eavesdropper's capacity to intercept sensitive data. This makes it difficult to secure the communication without inadvertently strengthening the eavesdropper's channel.

Nevertheless, physical layer security techniques can still ensure secure communication by exploiting the inherent randomness of wireless channels [12–16], even if the untrusted relay has access to critical information, such as the codebook [17]. These techniques can also mitigate the risks associated with jamming and eavesdropping, ensuring the confidentiality and integrity of transmitted data in the increasingly interconnected landscape of remote sensing and satellite communications [18, 19].

In cognitive radio (CR) hybrid satellite-terrestrial networks, the GU operates in the spectrum allocated to terrestrial primary users (PUs) and communicates with the satellites [20]. This spectrum sharing strategy is implemented to address the scarcity of available spectrum in satellite-terrestrial systems, allowing the GU to opportunistically access the same frequency bands as terrestrial PUs. However, this introduces additional challenges, as the GU must limit its transmit power to avoid causing interference to PUs, such as terrestrial base stations and mobile users. Failure to control the interference can degrade the communication quality for the PUs. Consequently, power control becomes a critical component in ensuring the delivery performance in both the primary and secondary networks, balancing the need for effective communication between the GU and the satellite, while protecting the quality of service for the terrestrial PUs. This power control constraint also makes it challenging for the GU to establish a direct link to the GEO satellite, especially in scenarios with heavy obstructions. In such cases, LEO satellites can effectively serve as relays, forwarding messages from the GU to the GEO satellite. Amplify-and-Forward (AF) relaying is particularly relevant in satellite systems due to its simplicity and efficiency in scenarios where the relay may not have the computational resources required for more complex strategies like Decode-and-Forward (DF) relaying [21]. However, the simplicity of AF relaying also makes it more vulnerable to security threats, as the relay simply amplifies and forwards everything it receives, including any potential noise or maliciously modified data [22–24].

In recent years, research has explored the physical layer security of communication systems with untrusted relays. For example, previous works have considered the secrecy performance of untrusted relays in terrestrial cooperative systems [25, 26] or cognitive radio networks [5, 27]. However, the application of these security concepts to cognitive satellite-terrestrial networks with untrusted LEO relays remains largely unexplored.

In this paper, we focus on analyzing the secrecy rate in a CR hybrid satellite-terrestrial network where an untrusted LEO satellite serves as an AF relay between a GU and a GEO satellite. In order to mitigate the eavesdropping threat posed by the untrusted LEO relay, a friendly LEO jammer transmits artificial noise to disrupt the relay's ability to intercept the communication. We derive closed-form expressions for the secrecy rate and investigate how different system parameters, such as channel fading, power control, and jamming, affect the physical layer security of the system. Our analysis is conducted within a CR framework, where the GU's transmit power is constrained by interference limits at terrestrial PUs. The key contributions of this paper

are outlined as follows:

- We develop an analytical model for the secrecy rate in a CR satellite-terrestrial network with an untrusted LEO AF relay and a friendly LEO jammer, considering various system parameters such as channel fading and power constraints imposed by CR.
- We derive an exact closed-form expression for the secrecy rate, providing insights into the security performance of the system under untrusted AF relaying.
- We validate the analytical results through Monte Carlo simulations and explore how power limitations imposed by CR, as well as the impact of jamming and shadowing, affect the secrecy rate.

The remainder of this paper is organized as follows: Section 2 introduces the system model, describing the untrusted LEO relay scenario and the cognitive spectrum sharing framework. In Section 3, we derive the closed-form expression for the secrecy rate under AF relaying. Section 4 presents the numerical results and validates the analytical expressions through simulations. Finally, Section 5 concludes the paper and discusses potential future research directions.

System Model

In this paper, we consider a cognitive satellite-terrestrial communication system, as shown in Fig 1 , where a GU transmits confidential data to a GEO satellite through an LEO satellite, which operates as an AF relay. The system operates under a CR framework, meaning that the GU uses the same spectrum as the terrestrial primary networks. In such an environment, the transmit power of the GU is constrained to ensure that the interference to the terrestrial primary users (PUs), remains below a specific threshold. A direct connection between the GU and the GEO satellite is not feasible due to heavy obstructions, such as buildings or terrain, causing significant shadowing. Additionally, the power control imposed by the underlay CR framework further challenges the feasibility of a direct GU-to-GEO link, as the GU must limit its transmit power to avoid causing interference to terrestrial PUs.

Fig 1. System Model. A cognitive satellite-terrestrial communication system model. See S1 Fig.1.

The LEO relay is not fully trusted to forward the message securely, and it may potentially eavesdrop on the transmitted messages. To mitigate this risk, we introduce a cooperative jamming strategy where another LEO satellite serves as a friendly jammer. This jammer is located outside the field-of-view of the GU, so it cannot act as a possible relay for forwarding the messages from the GU to the GEO satellite. However, it can still transmit interference to the untrusted LEO relay, effectively degrading the relay's ability to eavesdrop on the transmitted messages.

The message transmission from the GU to the GEO satellite occurs over two time slots. In the first time slot, the GU transmits its signal to the untrusted LEO satellite, while a friendly LEO jammer simultaneously sends artificial noise to the untrusted LEO satellite to degrade its eavesdropping capability. In the second time slot, the untrusted LEO satellite forwards the signal received from the GU in the first time slot to the GEO satellite, applying the AF relaying strategy.

We consider a widely used underlay CR framework, where the transmit power of the GU (denoted by P_U) is constrained to ensure that the interference at the PU remains

below a predefined threshold, I_{th}. The channel gain between the GU and the PU is represented by h_T, and the interference constraint is expressed as $P_U|h_T|^2 \leq I_{\text{th}}$, Additionally, we account for a maximum power constraint, denoted by P_M, at the GU. Therefore, the transmit power at the GU in this underlay CR network is given by (cf. [28])

$$P_U = \min\left\{\frac{I_{\text{th}}}{|h_T|^2}, P_M\right\}. \tag{1}$$

In contrast to the GU's power control imposed by the CR framework, the friendly LEO jammer operates without such power limitations, as it is far away from the terrestrial primary networks.

Communication Links

GU-to-LEO Link

The GU transmits a signal to the LEO satellite over a wireless link subject to Rician-Shadowed fading. The channel gain for this link is denoted as h_L, and follows a Rician-Shadowed distribution characterized by three parameters: the shape parameter m_0, the average power of the scattered component $2b$, and the power ratio between the line-of-sight (LOS) and scattered components, denoted by Ω. The probability density function (PDF) of the Rician-Shadowed fading channel power gain $|h_L|^2$ is given by the following expression [29]

$$f_{|h_L|^2}(x) = \alpha \exp\left(-\beta x\right) \ {}_1F_1\left(m_0; 1; \delta x\right), \tag{2}$$

where $\alpha = \frac{(2bm_0)^{m_0}}{2b(2bm_0+\Omega)^{m_0}}$, $\beta = \frac{1}{2b}$, and $\delta = \frac{\Omega}{2b(2bm_0+\Omega)}$ are functions of the Rician-Shadowed fading parameters m_0, b, and Ω, and ${}_1F_1(\cdot)$ is the confluent hypergeometric function of the first kind. For simplification, we consider that m_0 is a positive integer. Then, the PDF and the cumulative distribution function (CDF) of $|h_L|^2$ are respectively of the form [30]

$$f_{|h_L|^2}(x) = \alpha \sum_{i=0}^{m_0-1} \zeta(i) x^i \exp(-(\beta-\delta)x), \tag{3}$$

$$F_{|h_L|^2}(x) = 1 - \alpha \sum_{i=0}^{m_0-1} \frac{\zeta(i)}{(\beta-\delta)^{i+1}} \Gamma\left(i+1, (\beta-\delta)x\right), \tag{4}$$

where $\zeta(i) \triangleq \binom{m_0-1}{i}\frac{\delta^i}{i!}$, and $\Gamma(\cdot,\cdot)$ denotes the upper incomplete Gamma function.

Satellite-to-Satellite Links

The LEO satellite forwards the amplified signal to the GEO satellite. The LEO-to-GEO link is considered to be subject only to large-scale path loss, and the channel gain for this link is denoted as L_G, representing the path loss. Satellite-to-satellite links typically do not experience small-scale fading due to the absence of obstacles in space and the direct LoS nature of the communications, which eliminates the effects of multipath propagation. The signal received at the GEO satellite is given by

$$y_G = \sqrt{L_G} x_L + n_G, \tag{5}$$

where x_L is the amplified signal sent from the LEO satellite, and n_G is the Additive White Gaussian Noise (AWGN) at the GEO satellite with the variance of σ_G^2. Similar to the LEO-to-GEO link, we assume that the link between the LEO jammer and the untrusted LEO relay experiences only large-scale path loss (denoted by L_J), with no small-scale fading effects.

GU-to-PU Link

For the GU-to-PU link, we assume a Nakagami-m fading channel. The PDF and CDF of $|h_T|^2$, with a mean of $m\theta$, are expressed as follows

$$f_{|h_T|^2}(x) = \frac{1}{\Gamma(m)\theta^m} x^{m-1} \exp\left(-\frac{x}{\theta}\right), \tag{6}$$

$$F_{|h_T|^2}(x) = \frac{1}{\Gamma(m)} \Upsilon\left(m, \frac{x}{\theta}\right), \tag{7}$$

where $\Gamma(\cdot)$ represents the Gamma function, and $\Upsilon(\cdot,\cdot)$ denotes the lower incomplete Gamma function.

Untrusted AF Relaying

The untrusted LEO satellite employs the AF relaying strategy, in which it amplifies the received signal from the GU and forwards it to the GEO satellite. The amplification gain at the LEO satellite is denoted by G_{AF}, and the signal received at the GEO satellite after relaying is

$$y_G = \sqrt{L_G} G_{\mathrm{AF}} \left(\sqrt{P_U} h_L x_U + n_L + \sqrt{L_J} z\right) + n_G, \tag{8}$$

where x_U is the transmitted signal from the GU, G_{AF} is the AF gain, and n_L is the AWGN with the variance of σ_L^2 at the LEO satellite. In the above, $z \sim \mathcal{CN}(0, \sigma_Z^2)$ represents the artificial noise transmitted by the friendly LEO jammer. Notably, the artificial noise z is known to the GEO satellite, whereas the untrusted LEO relay has no prior knowledge of it.

Since the LEO relay is untrusted, the GU does not perform channel training with the LEO satellite, meaning that the LEO satellite lacks knowledge of the instantaneous channel state information (CSI) for the GU-to-LEO link. This approach prevents the untrusted LEO relay from adapting its gain to degrade the SNR at the GEO satellite. In this scenario, a fixed amplification factor is considered, as the LEO satellite does not have the instantaneous CSI of the first hop. The fixed AF relay gain ensures that the LEO relay maintains a consistent level of amplification regardless of the instantaneous channel conditions between the GU and the LEO satellite. By fixing the relay gain, the system maintains control over potential security risks posed by the untrusted relay, while still enabling the LEO satellite to forward the signal to the GEO satellite.

Since the GU does not engage in channel training with the LEO satellite, and due to the absence of a direct transmission path between the GU and the GEO satellite caused by significant LoS obstructions, the GEO satellite lacks access to the instantaneous CSI for both the GU-LEO link and the GU-terrestrial PU link. As a result, the GEO satellite demodulates the received signal based solely on the statistical properties of the GU-LEO channel and the GU-PU channel. This reliance on statistical knowledge complicates the signal decoding process, as the GEO cannot utilize instantaneous CSI to optimize its decoding strategy. Instead, the GEO must rely on the channel's average behavior to decode the signal. Given this scenario and removing the artificial noise, the signal for decoding x_U is of the form

$$y'_G = \sqrt{L_G} G_{\mathrm{AF}} \left(\sqrt{P_U} h_L x_U + n_L\right) + n_G, \tag{9}$$

which can be re-written as (10), where $\mathbb{E}\{\cdot\}$ denotes the expectation operator.

$$\begin{aligned} y'_G = &\sqrt{L_G} G_{\mathrm{AF}} \mathbb{E}\{\sqrt{P_U}|h_L|\} x_U \\ &+ \sqrt{L_G} G_{\mathrm{AF}} (\sqrt{P_U} h_L - \mathbb{E}\{\sqrt{P_U}|h_L|\}) x_U \\ &+ \sqrt{L_G} G_{\mathrm{AF}} n_L + n_G. \end{aligned} \tag{10}$$

The end-to-end SNR at the GEO satellite, denoted as γ_G, can be expressed as

$$\gamma_G = \frac{L_G G_{\mathrm{AF}}^2 \mathbb{E}^2\{\sqrt{P_U}|h_L|\}}{\sigma_G^2 + L_G G_{\mathrm{AF}}^2 \sigma_L^2 + L_G G_{\mathrm{AF}}^2 \mathrm{Var}\{\sqrt{P_U} h_L\}}, \tag{11}$$

where $\mathrm{Var}\{\cdot\}$ denotes the variance of the inside random variable.

To decode the signal sent from the GU based on the statistical CSI knowledge, the received signal at the untrusted LEO satellite can be rewritten as

$$\begin{aligned} y_L &= \mathbb{E}\{\sqrt{P_U}|h_L|\} x_U \\ &\quad + \left(\sqrt{P_U} h_L - \mathbb{E}\{\sqrt{P_U}|h_L|\}\right) x_U \\ &\quad + \sqrt{L_J} z + n_L. \end{aligned} \tag{12}$$

The SNR for decoding x_U at the LEO satellite is of the form

$$\gamma_L = \frac{\mathbb{E}^2\{\sqrt{P_U}|h_L|\}}{L_J \sigma_Z^2 + \sigma_L^2 + \mathrm{Var}\{\sqrt{P_U}|h_L|\}}. \tag{13}$$

Secrecy Rate

We aim to analyze the secrecy rate of the communication link between the GU and the GEO satellite in the presence of both an untrusted LEO relay and a friendly LEO jammer. The secrecy rate is defined as the difference between the achievable rates at the GEO satellite and the LEO relay. This rate represents the secure transmission capacity of the information sent from the GU to the GEO satellite.

Mathematically, the secrecy rate C_s is expressed as

$$C_s = \frac{1}{2}\Big[\log_2(1+\gamma_G) - \log_2(1+\gamma_L)\Big]^+, \tag{14}$$

where γ_G is the SNR at the GEO satellite and γ_L is the SNR at the untrusted LEO relay. The term $[x]^+ = \max\{x, 0\}$ ensures that the secrecy rate is non-negative. The friendly jammer reduces γ_L, thereby improving the secrecy rate by degrading the LEO relay's ability to intercept the message.

Performance Analysis

In this section, we will derive the closed-form expression for the secrecy rate. Observing (11), (13) and (14), we know that we should derive the closed-form expressions for $\mathbb{E}\{\sqrt{P_U}|h_L|\}$ and $\mathrm{Var}\{\sqrt{P_U}|h_L|\}$.

Theorem 1. *In the presence of an untrusted LEO relay and an LEO jammer, the secrecy rate of the considered hybrid cognitive hybrid terrestrial-satellite networks takes the form in* (14) *with the expressions for* $\mathbb{E}\{\sqrt{P_U}|h_L|\}$ *and* $\mathrm{Var}\{\sqrt{P_U}|h_L|\}$ *respectively given by*

$$\mathbb{E}\{\sqrt{P_U}|h_L|\} = \mathbb{E}\{\sqrt{P_U}\}\mathbb{E}\{|h_L|\} \tag{15}$$

$$\mathrm{Var}\{\sqrt{P_U}|h_L|\} = \mathbb{E}\{P_U\}\mathbb{E}\{|h_L|^2\} - \mathbb{E}^2\{\sqrt{P_U}\}\mathbb{E}^2\{|h_L|\} \tag{16}$$

where the mean of $\sqrt{P_U}$ *is given by* (17).

$$\mathbb{E}\{\sqrt{P_U}\} = \frac{\sqrt{P_M}}{\Gamma(m)} \Upsilon\left(m, \sqrt{\frac{I_{\mathrm{th}}}{P_M \theta^2}}\right) + \frac{\sqrt{I_{\mathrm{th}}/\theta}}{\Gamma(m)} \Gamma\left(m - 0.5, \frac{I_{\mathrm{th}}}{P_M \theta}\right) \tag{17}$$

The closed-form expressions for $\mathbb{E}\{\sqrt{P_U}\}$, $\mathbb{E}\{|h_L|\}$ *and* $\mathbb{E}\{|h_L|^2\}$ *are respectively*

$$\mathbb{E}\{P_U\} = \frac{P_M}{\Gamma(m)}\Upsilon\left(m, \frac{I_{\rm th}}{P_M\theta}\right) + \frac{I_{th}/\theta}{\Gamma(m)}\Gamma\left(m-1, \frac{I_{\rm th}}{P_M\theta}\right), \tag{18}$$

$$\mathbb{E}\{|h_L|\} = \alpha\sum_{i=0}^{m_0-1}\zeta(i)\frac{\Gamma(i+1.5)}{(\beta-\delta)^{i+1.5}}, \tag{19}$$

$$\mathbb{E}\{|h_L|^2\} = 2b+\Omega. \tag{20}$$

Proof of Theorem 1. For $\mathbb{E}\{\sqrt{P_U}\}$, we have that

$$\begin{aligned}
\mathbb{E}\{\sqrt{P_U}\} &= \int_0^\infty \sqrt{P_U} f_{|h_T|^2}(x)\mathrm{d}x \\
&= \int_0^\infty \min\left\{\sqrt{\frac{I_{\rm th}}{x}}, \sqrt{P_M}\right\} f_{|h_T|^2}(x)\mathrm{d}x \\
&= \int_0^{I_{\rm th}/P_M} \sqrt{P_M} f_{|h_T|^2}(x)\mathrm{d}x + \int_{I_{\rm th}/P_M}^\infty \sqrt{\frac{I_{th}}{x}} f_{|h_T|^2}(x)\mathrm{d}x \\
&= \sqrt{P_M}F_{|h_T|^2}\left(\frac{I_{\rm th}}{P_M}\right) + \int_{I_{\rm th}/P_M}^\infty \sqrt{\frac{I_{th}}{x}} f_{|h_T|^2}(x)\mathrm{d}x.
\end{aligned} \tag{21}$$

For the second term in the above, we have that

$$\begin{aligned}
\int_{I_{\rm th}/P_M}^\infty \sqrt{\frac{I_{th}}{x}} f_{|h_T|^2}(x)\mathrm{d}x &= \frac{\sqrt{I_{\rm th}}}{\Gamma(m)\theta^m}\int_{I_{\rm th}/P_M}^\infty x^{m-1.5}\exp\left(-\frac{x}{\theta}\right)\mathrm{d}x \\
&\overset{(a)}{=} \frac{\sqrt{I_{\rm th}/\theta}}{\Gamma(m)}\Gamma\left(m-0.5, \frac{I_{\rm th}}{P_M\theta}\right)
\end{aligned} \tag{22}$$

where (a) follows from [31, Eq. (3.381.3)].

For the mean of P_U, we have that

$$\begin{aligned}
\mathbb{E}\{P_U\} &= \int_0^\infty \min\left\{\frac{I_{\rm th}}{x}, P_M\right\} f_{|h_T|^2}(x)\mathrm{d}x \\
&= \int_0^{I_{\rm th}/P_M} P_M f_{|h_T|^2}(x)\mathrm{d}x + \int_{I_{\rm th}/P_M}^\infty \frac{I_{th}}{x} f_{|h_T|^2}(x)\mathrm{d}x \\
&= P_M F_{|h_T|^2}\left(\frac{I_{\rm th}}{P_M}\right) + \int_{I_{\rm th}/P_M}^\infty \frac{I_{th}}{x} f_{|h_T|^2}(x)\mathrm{d}x
\end{aligned} \tag{23}$$

where

$$\begin{aligned}
\int_{I_{\rm th}/P_M}^\infty \frac{I_{th}}{x} f_{|h_T|^2}(x)\mathrm{d}x &= \frac{I_{th}}{\Gamma(m)\theta^m}\int_{I_{\rm th}/P_M}^\infty x^{m-2}\exp(-x/\theta)\mathrm{d}x \\
&\overset{(a)}{=} \frac{I_{th}/\theta}{\Gamma(m)}\Gamma\left(m-1, \frac{I_{\rm th}}{P_M\theta}\right),
\end{aligned} \tag{24}$$

where (a) follows from [31, Eq. (3.381.3)]. Combining (23) and (24) yields (18).

For $\mathbb{E}\{|h_L|\}$, by using the PDF of $|h_L|^2$, we have that

$$\begin{aligned}
\mathbb{E}\{|h_L|\} &= \alpha\sum_{i=0}^{m_0-1}\zeta(i)\int_0^\infty x^{i+0.5}\exp(-(\beta-\delta)x)\mathrm{d}x \\
&\overset{(a)}{=} \alpha\sum_{i=0}^{m_0-1}\zeta(i)\frac{\Gamma(i+1.5)}{(\beta-\delta)^{i+1.5}}
\end{aligned} \tag{25}$$

where (a) follows from [31, Eq. (3.381.4)]. Referring to [32, Prop. 5.1], we know that

$$\mathbb{E}\{|h_L|^2\} = 2b + \Omega. \tag{26}$$

We conclude the proof. □

Numerical Results and Discussion

In this section, we present the numerical results for the secrecy rate in the considered cognitive satellite-terrestrial network with an untrusted LEO relay and a friendly LEO jammer. The results are validated through Monte Carlo simulations, and we analyze the impact of various system parameters on the secrecy rate. For simplicity, we set $L_J = L_G = \sigma_G^2 = \sigma_L^2 = \theta = 1$. In Figures 2 - 4 , the GU-to-LEO relay link is subject to infrequent light shadowing, with parameters $m_0 = 20$, $b = 0.158$, and $\Omega = 1.29$ (cf. [29, Table III]). In Fig 5, following [29, Table III], we examine three different shadowing conditions for the GU-to-LEO relay link: frequent heavy shadowing ($m_0 = 1$, $b = 0.063$, and $\Omega = 8.97 \times 10^{-4}$), average shadowing ($m_0 = 10$, $b = 0.126$, and $\Omega = 0.835$), and infrequent light shadowing ($m_0 = 20$, $b = 0.158$, and $\Omega = 1.29$).

Fig 2 illustrates the impact of the interference threshold I_{th} on the secrecy rate under different artificial noise power levels. As I_{th} increases, the secrecy rate improves, as a higher threshold allows the GU to transmit with greater power, enhancing the SNR at the GEO satellite. However, beyond a certain point, the secrecy rate stabilizes and becomes nearly unchanged. This is due to the maximum transmit power constraint at the GU, which limits the GU's ability to further increase its power, regardless of the increase in I_{th}. Therefore, once the GU reaches its maximum transmit power, further increases in I_{th} provide no additional benefit. This indicates that the system is bound by the maximum power limit of the GU, making the adjustment of I_{th} effective only up to a certain level. Beyond that, the secrecy rate plateaus, and the system performance depends more on other factors such as the artificial noise power σ_Z^2.

Fig 2. Secrecy rate versus I_{th} with $P_M = 10$ dB, $G_{\text{AF}} = 1$, $m = 2$, $m_0 = 20$, $b = 0.158$, and $\Omega = 1.29$. The impact of the interference threshold I_{th} on the secrecy rate under different artificial noise power levels. See S1 Fig.2.

In Fig 3, we remove the maximum transmit power constraint, allowing the GU to transmit with power always close to $I_{\text{th}}/|h_T|^2$. Under this setting, the secrecy rate initially increases with I_{th}, reaching a peak, and then decreases. The initial increase occurs because a higher I_{th} enhances the link quality between the GU and the GEO satellite, improving the achievable rate at the GEO satellite. However, as I_{th} continues to increase, the untrusted LEO relay's ability to intercept the message improves as well, because the influence of the artificial noise σ_Z^2 becomes less significant. When I_{th} is sufficiently large, the artificial noise becomes almost ineffective at hindering the untrusted relay, leading to a decrease in the secrecy rate. This suggests that there is an optimal value of I_{th} that balances the improvement in the legitimate link and the negative effect on the eavesdropping link. The value of I_{th} can be adjusted by varying the distance between the GU and the PU, which is a crucial factor in determining the overall secrecy performance.

Fig 3. Secrecy rate versus I_{th} with $P_M \to \infty$, $G_{\text{AF}} = 1$, $\sigma_Z^2 = 5$, $m_0 = 20$, $b = 0.158$, and $\Omega = 1.29$. See S1 Fig.3.

Fig 4 examines the effect of the AF gain G_{AF} on the secrecy rate. As G_{AF} increases, the secrecy rate also improves because a higher AF gain amplifies the received signal at

the GEO satellite, leading to a better end-to-end SNR. However, as G_{AF} becomes sufficiently large, further increases no longer result in noticeable improvements in the secrecy rate. This is due to the fact that a higher AF gain also amplifies the noise in the first hop (GU-to-LEO link). As a result, while the legitimate signal is amplified, the accompanying noise is also magnified, which limits the benefit of increasing G_{AF} indefinitely. The results indicate that although a moderate AF gain can significantly improve the secrecy rate, there is a saturation point beyond which the noise amplification "equals" the signal amplification.

Fig 4. Secrecy rate versus I_{th} with $P_M = 10$ dB, $m = 2$, $m_0 = 20$, $b = 0.158$, and $\Omega = 1.29$. See S1 Fig.4.

Fig 5 shows the secrecy rate under three different shadowing conditions for the GU-to-LEO link: frequent heavy shadowing, average shadowing, and infrequent light shadowing. As expected, lighter shadowing leads to higher SNRs at both the untrusted LEO relay and the GEO satellite. While lighter shadowing improves the decoding capability of the untrusted relay, it also benefits the legitimate link to the GEO satellite by improving the end-to-end SNR. Interestingly, the results imply that lighter shadowing is generally favorable for secure transmission, as the overall improvement in the end-to-end link dominates the potential advantage gained by the untrusted relay. Therefore, despite the improvement in the eavesdropper's channel quality, the GEO satellite's ability to decode the confidential message is enhanced more substantially under lighter shadowing conditions, leading to better secrecy performance.

Fig 5. Secrecy rate versus I_{th} with $P_M = 10$ dB, $G_{\mathrm{AF}} = 1$, $m = 2$ and $\sigma_Z^2 = 5$. See S1 Fig.5.

In conclusion, the numerical results reveal that key parameters such as the interference threshold I_{th}, AF gain G_{AF}, and the shadowing conditions play significant roles in determining the secrecy rate. Adjusting these parameters can help balance the improvement in the legitimate link and the impact of eavesdropping, providing valuable insights into how to optimize the system's secrecy performance in the presence of an untrusted LEO relay and a friendly LEO jammer. Moreover, the excellent agreement between the simulated results and the theoretical analysis in Figs. 2–5 fully validates the accuracy and reliability of the developed analytical model, confirming its robustness in predicting system behavior under various conditions.

Conclusions

In this paper, we analyzed the physical layer security of a cognitive hybrid satellite-terrestrial communication system where a ground user communicates with a GEO satellite through an untrusted LEO relay, with the presence of a friendly LEO jammer. We derived the closed-form expression for the secrecy rate and investigated the impact of key system parameters, such as the interference threshold, AF gain, and shadowing effects, on the secrecy performance. Our results show that increasing the interference threshold improves the secrecy rate up to a certain limit, after which the impact of the untrusted relay diminishes the overall security. The AF gain was also found to improve secrecy but with diminishing returns as the noise amplification limits its benefits. Lastly, our analysis revealed that lighter shadowing conditions, despite improving the untrusted relay's channel, generally favor secure communication by significantly enhancing the end-to-end SNR at the GEO satellite.

The findings highlight the importance of carefully balancing system parameters to optimize security in cognitive satellite-terrestrial networks. Future work can explore further optimizations, such as power allocation strategies and advanced jamming techniques, to enhance the physical layer security in these systems.

Supporting information

S1 Fig.1 System Model. The figure file name is: System_Model.tif.

S1 Fig.2 Secrecy rate versus I_{th} with $P_M = 10$ dB, $G_{\text{AF}} = 1$, $m = 2$, $m_0 = 20$, $b = 0.158$, and $\Omega = 1.29$. The figure file name is: SecrecyRate_Ith.tif

S1 Fig.3 Secrecy rate versus I_{th} with $P_M \to \infty$, $G_{\text{AF}} = 1$, $\sigma_Z^2 = 5$, $m_0 = 20$, $b = 0.158$, and $\Omega = 1.29$. The figure file name is: SecrecyRate_Ith_m.tif.

S1 Fig.4 Secrecy rate versus I_{th} with $P_M = 10$ dB, $m = 2$, $m_0 = 20$, $b = 0.158$, and $\Omega = 1.29$. The figure file name is: SecrecyRate_Ith_G.tif.

S1 Fig.5 Secrecy rate versus I_{th} with $P_M = 10$ dB, $G_{\text{AF}} = 1$, $m = 2$ and $\sigma_Z^2 = 5$. The figure file name is: SecrecyRate_Ith_Shadow.tif.

Acknowledgments

The work was supported in part by the National Natural Science Foundation of China under Grant No. 61471269 and No. 61801266, and the Science and Technology Development Plan Project of Weifang City under Grant No. 2024GX027.

References

1. Ma T, Qian B, Qin X, et al. Satellite-terrestrial integrated 6G: an ultra-dense LEO networking management architecture. IEEE Wireless Communications. 2024 Feb; 31(1):62 - 69.

2. He Q, Xiang Z, Ren P. A coordinated non-orthogonal multiple access strategy for integrated terrestrial-satellite networks. PLoS ONE. 2021 Mar; 16(3): e0248173.

3. Hosseinian M, Choi JP, Chang SH, et al. Review of 5G NTN standards development and technical challenges for satellite integration with the 5G network. IEEE Aerospace and Electronic Systems Magazine. 2021 Aug; 36(8):22-31.

4. Feng BH, Li GL, Li GW,et al.. Enabling efficient service function chains at terrestrial-satellite hybrid cloud networks. IEEE Network. 2019 Nov-Dec; 33(6):94-99.

5. Tirmizi SBR, Chen YF, Lakshminarayana S, Feng W and Khuwaja AA. Hybrid satellite–terrestrial networks toward 6G: key technologies and open issues. Sensors. 2022 Nov; 22(21):8544.

6. Wang X, Lin XJ, Jia M. Spatial Information Entropy-Assisted Integrated Sensing and Communication for Integrated Satellite-Terrestrial Networks. Electronics.2024 Aug; 13(15):3082.

7. Wang RB, Talgat A, Kishk MA, Alouini MS. Conditional contact angle distribution in LEO satellite-relayed transmission. IEEE Communications Letters. 2022 Nov; 26(11):2735-2739.

8. Lin XH, Zhang HX, Pan GF, Wang S, An JP. LEO relay-aided GEO satellite-terrestrial transmissions. IEEE Transactions on Vehicular Technology. 2023 Dec; 72(12):16899-16904.

9. Dong FH, Song JX, Zhang YS, Wang YQ, Huang T. DRL-Based load-balancing routing scheme for 6G space–air–ground integrated networks. Remote Sensing. 2023 May; 15(11):2801.

10. Liu MY, Tu R, Chen QS, Li Q, Chen JM, Zhang PF, Lu XC. Study of fast and reliable time transfer methods using low earth orbit enhancement. Remote Sensing. 2024 Jun; 16(11):2044.

11. Çelikbilek K, Saleem Z, Morales FR, Praks J, Lohan ES. Survey on optimization methods for LEO-satellite-based networks with applications in future autonomous transportation. Sensors. 2022 Feb; 22(4):1421.

12. Bloch M, Barros J, Rodrigues MRD, Mclaughlin SW. Wireless information-theoretic security. IEEE Transactions on Information Theory. 2008 Jun; 54(6):2515-2534.

13. Zhao H, Tan Y, Pan G, Chen Y, Yan N. Secrecy outage on transmit antenna selection/maximal ratio combining in MIMO cognitive radio networks. IEEE Transactions on Vehicular Technology. 2016 Dec; 65(12):10236-10242.

14. Zhao H, Liu Y, Sultan-Salem A, Alouini MS. A simple evaluation for the secrecy outage probability over generalized-K fading channels. IEEE Communications Letters. 2019 Sep; 23(9):1479-1483.

15. Zhao H, Liu Z, Yang L, Alouini MS. Secrecy analysis in DF relay over generalized-K fading channels. IEEE Transactions on Communications. 2019 Oct; 67(10):7168-7182.

16. Wu ZK, Guo KF, Zhu SB. Covert communication for integrated satellite–terrestrial relay networks with cooperative jamming. Electronics. 2023 Feb; 12(4): 999.

17. Mekkawy T, Yao RG, Qi N, Lu YN. Secure relay selection for two way amplify-and-forward untrusted relaying networks. IEEE Transactions on Vehicular Technology. 2018 Dec; 67(12):11979-11987.

18. Chan S, Kim S, Oh KD. Energy-efficient physical layer security schemes for low Earth orbit satellite systems. International Journal of Satellite Communications and Networking. 2024 Sep-Oct; 42(5):374-396.

19. Abdallah W. A physical layer security scheme for 6G wireless networks using post-quantum cryptography. Computer Communications. 2024 Mar; 218:176-187.

20. Li B, Park J, Choi J. On optimal power control for cognitive satellite-terrestrial systems. IEEE Wireless Communications Letters. 2024 Oct; 13(10):2737-2741.

21. An K, Lin M, Liang T. On the performance of multiuser hybrid satellite-terrestrial relay networks with opportunistic scheduling. IEEE Communications Letters. 2015 Oct; 19(10):1722-1725.

22. Wang L, Cai YM, Zou YL, Yang WW, Hanzo L. Joint relay and jammer selection improves the physical layer security in the face of CSI feedback delays. IEEE Transactions on Vehicular Technology. 2016 Aug; 65(8):6259-6274.

23. Souzani A, Pourmina MA, Azmi P, Naser-Moghadasi M. Physical layer security enhancement via IRS based on PD-NOMA and cooperative jamming. cIEEE Access. 2023; 11:65956-65967.

24. Wang D, Bai B, Chen W, Han Z. Achieving high energy efficiency and physical-Layer security in AF relaying. IEEE Transactions on Wireless Communications. 2016 Jan; 15(1):740-752.

25. Lv L, Zhou FH, Chen J, Al-Dhahir N. Secure cooperative communications with an untrusted relay: a NOMA-inspired jamming and relaying approach. IEEE Transactions on Information Forensics and Security. 2019 Dec; 14(12):3191-3205.

26. Wang DW, Wu MH, He YX, Pang LN, Xu Q, Zhang RN. An HAP and UAVs collaboration framework for uplink secure rate maximization in NOMA-enabled IoT networks. Remote Sensing. 2022 Sep; 14(18):4501.

27. Chen DC, Cheng YP, Yang WW, Hu JW, Cai YM. Physical layer security in cognitive untrusted relay networks. IEEE Access. 2018; 6:7055-7065.

28. Zhao H, Zhang J, Yang L, Pan G, Alouini MS. Secure mmWave communications in cognitive radio networks. IEEE Wireless Communications Letters. 2019 Aug; 8(4):1171-1174.

29. Abdi A, Lau WC, Alouini MS, Kaveh M. A new simple model for land mobile satellite channels: first and second order statistics. IEEE Transactions on Wireless Communications. 2003 May; 2(3):519-528.

30. Zhao H, Bazco-Nogueras A, Elia P. Coded caching in land mobile satellite systems. Proc. IEEE Int. Conf. Commun. (ICC). 2022 May; Seoul, Korea.

31. Gradshteyn, Izrail S, Ryzhik, Iosif M. Table of integrals, series, and products. Seventh. Academic press: San Diego, C.A., USA, 2007.

32. Zhao H. High performance cache-aided downlink systems: novel algorithms and analysis. PHD Thesis. Sorbonne University, Paris, France, 2022.

Fig1

Click here to access/download;Figure;System_Model.tif

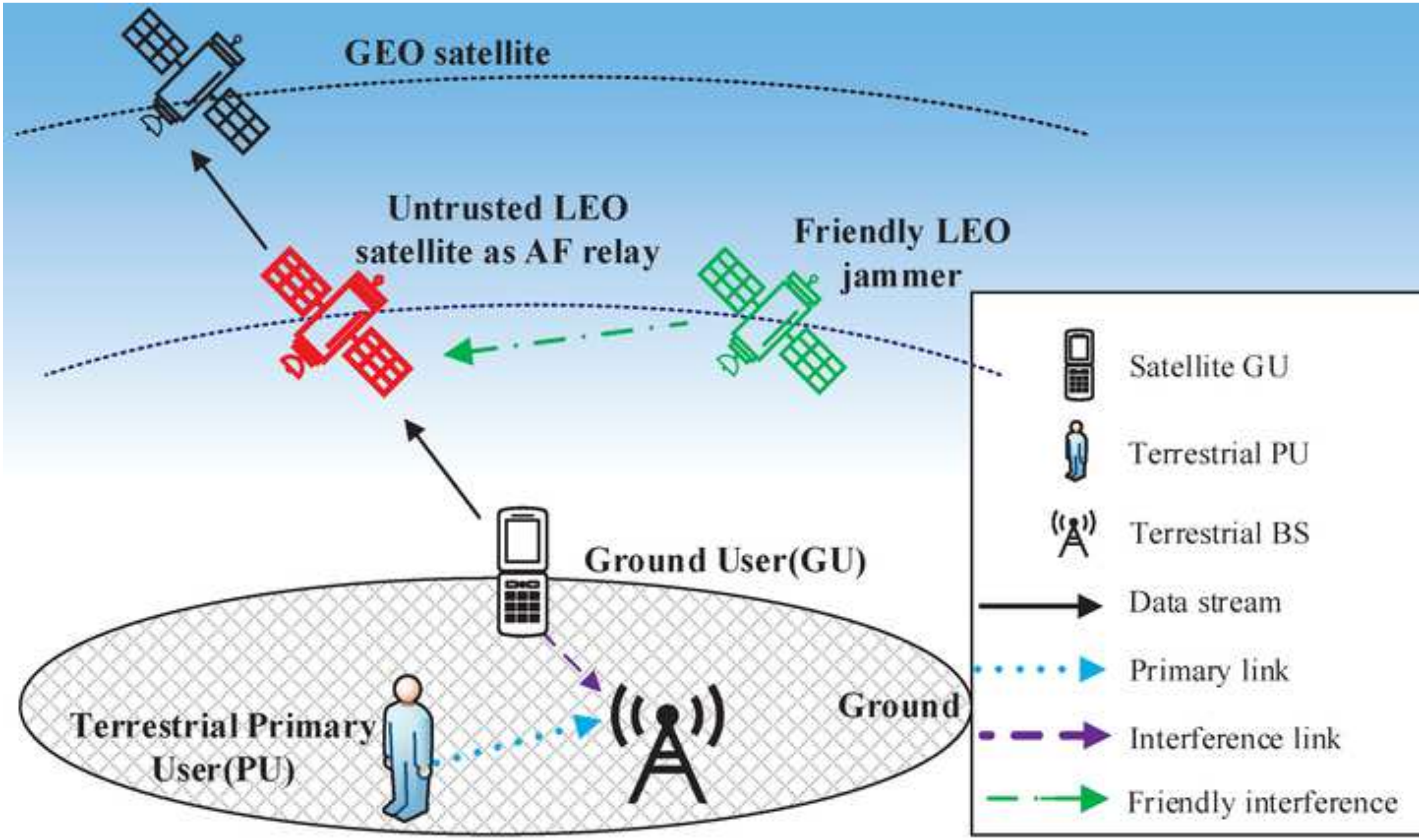

Fig2

Click here to access/download;Figure;SecrecyRate_Ith.tif

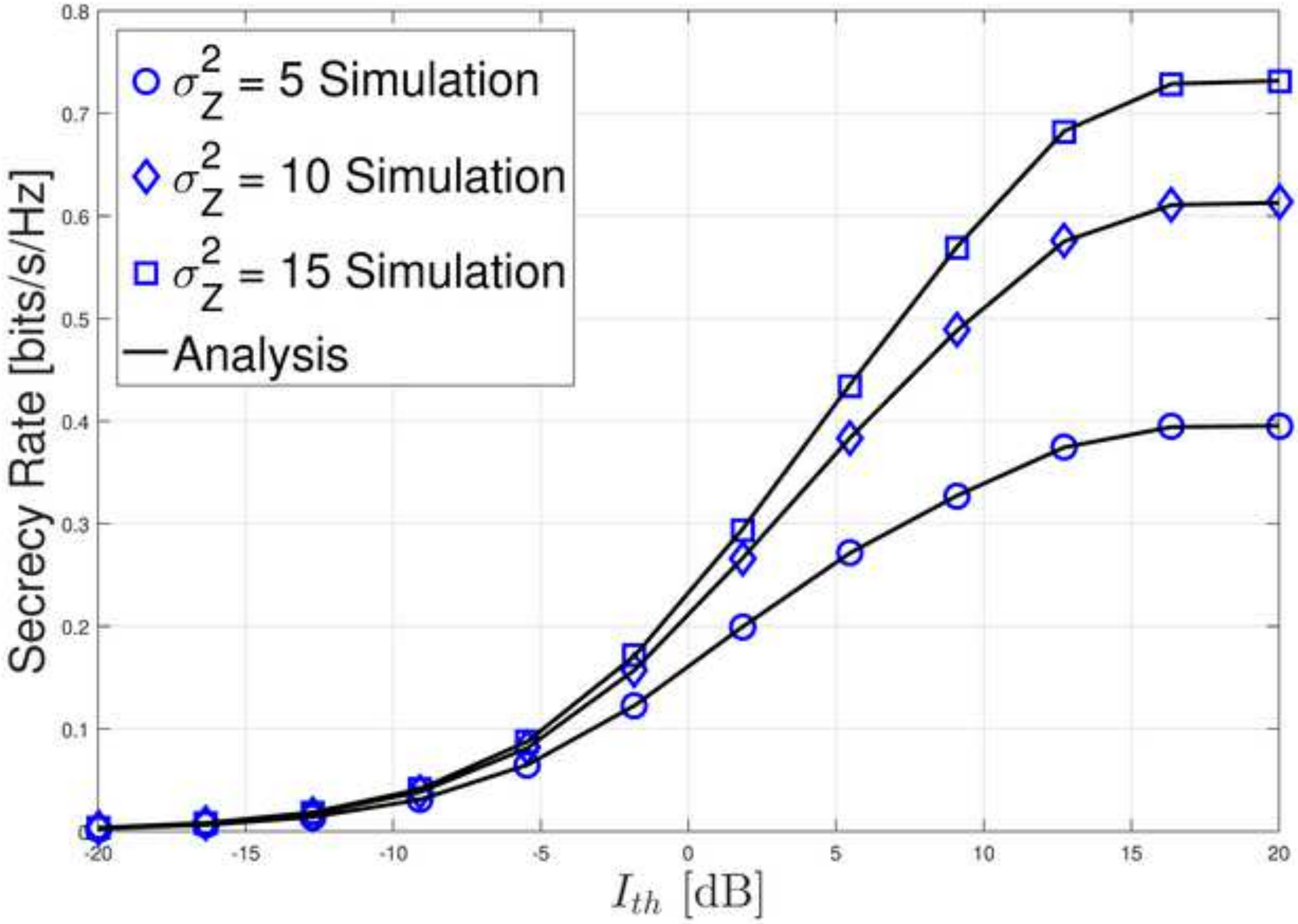

Fig3

Click here to access/download;Figure;SecrecyRate_Ith_m.tif

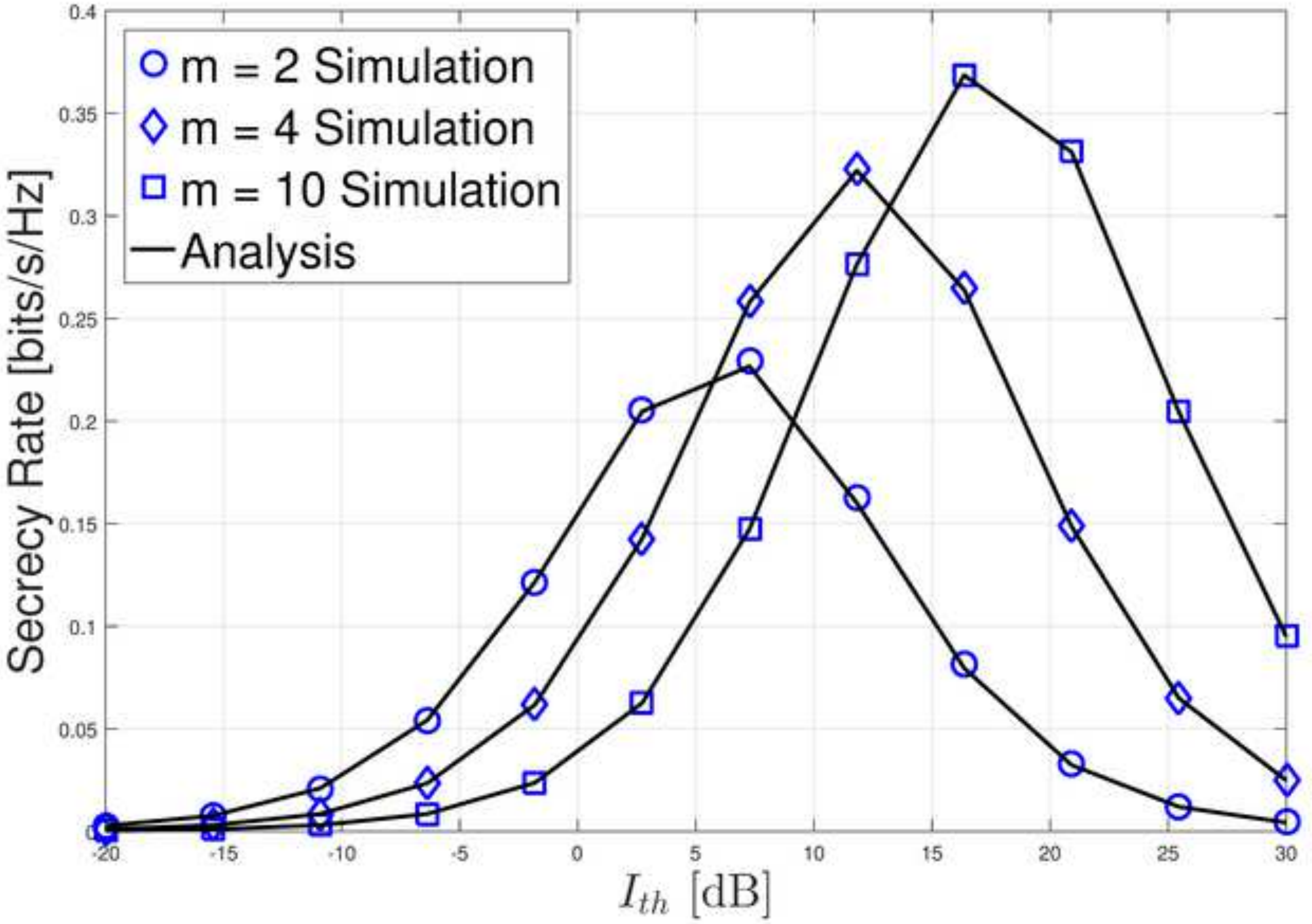

Fig4

Click here to access/download;Figure;SecrecyRate_Ith_G.tif

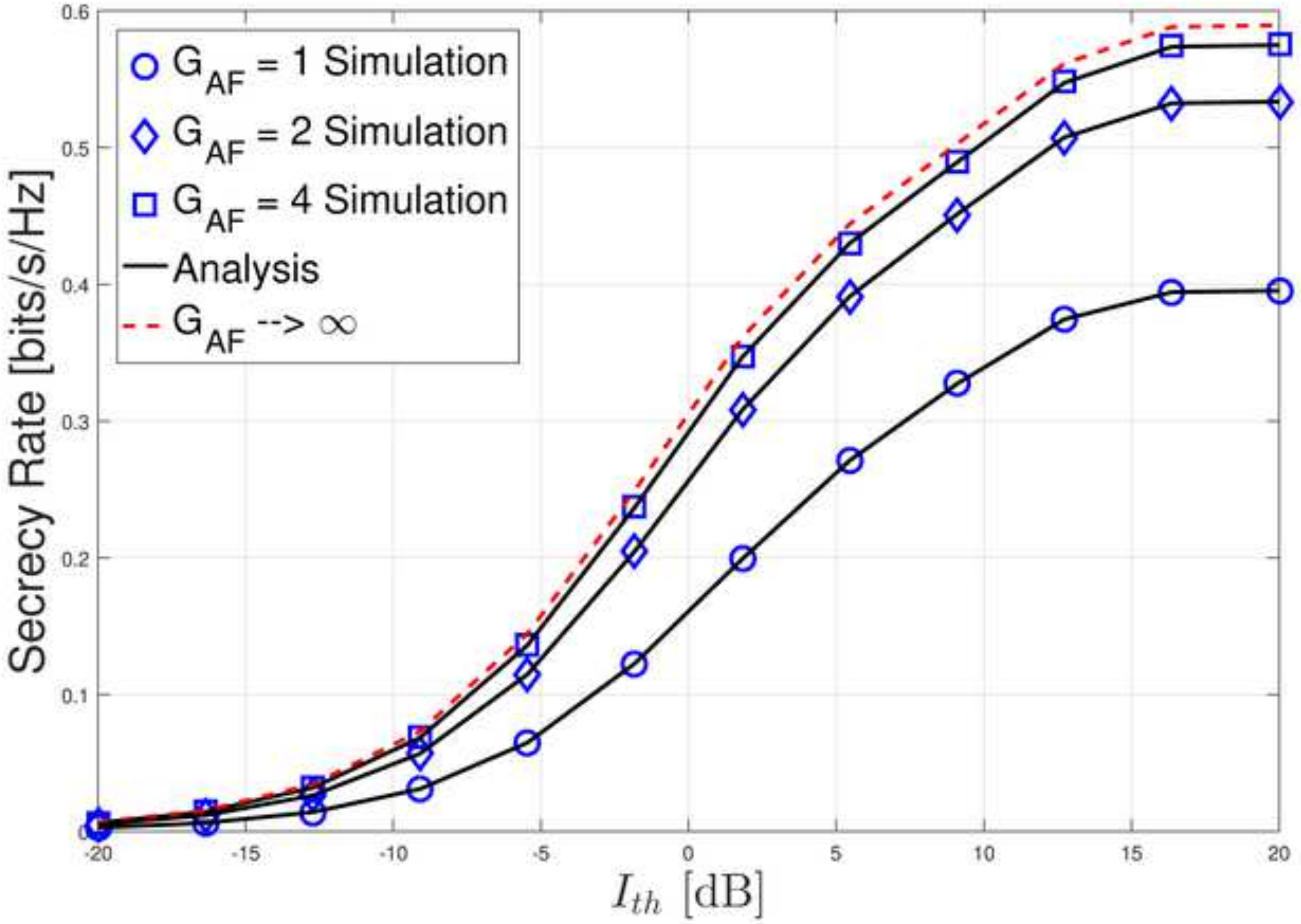

Fig5

Click here to access/download;Figure;SecrecyRate_Ith_Shadow.tif

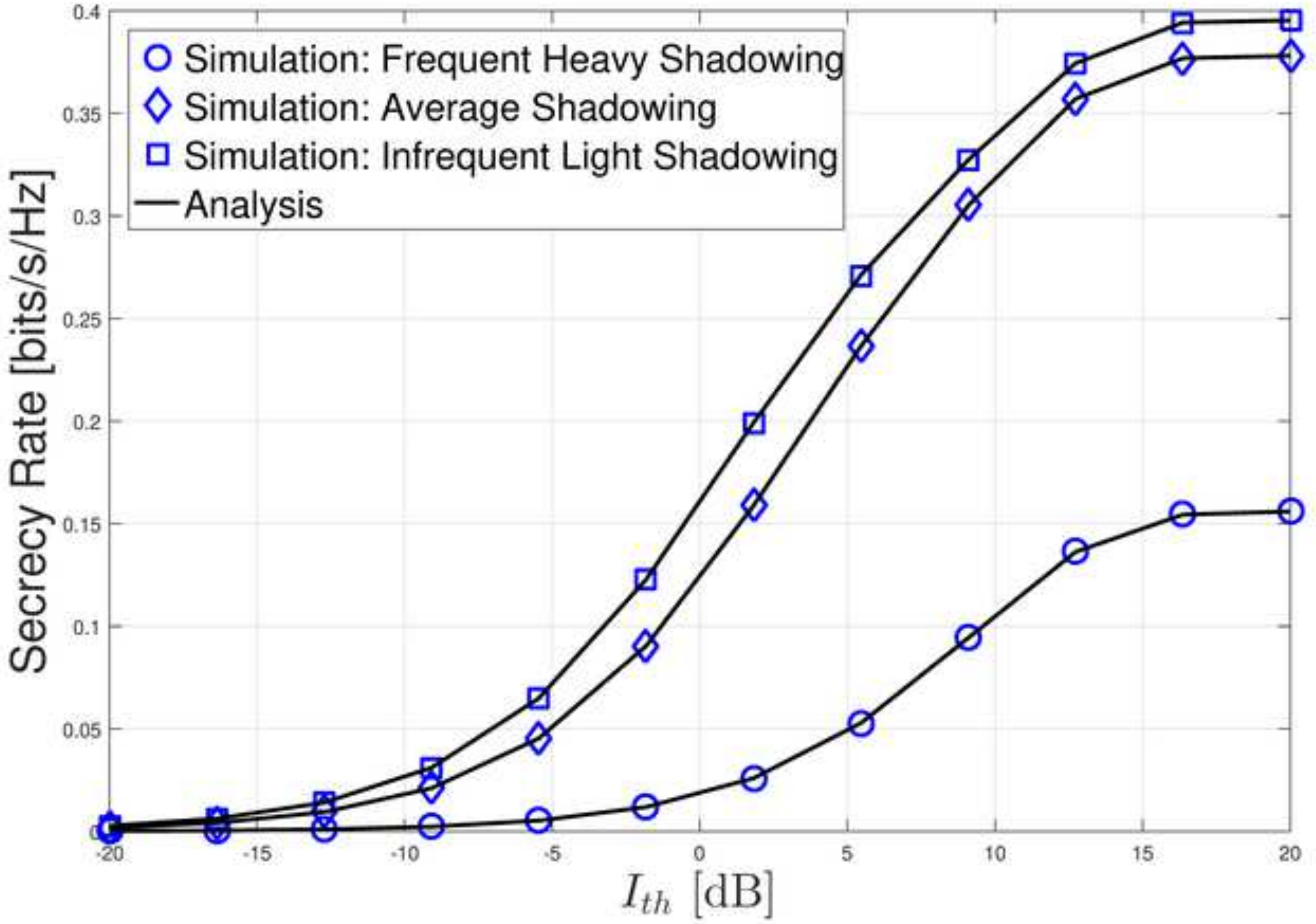

Supplement: Attachment — Submitted filename: PONE-D-25-16284_Suggestions and comments.pdf [file pone.0328729.s001.pdf]
