# Supplementary material for: Secure analysis in cognitive satellite-terrestrial networks with untrusted LEO relaying and friendly jamming
Source: PLoS One. 2025 Aug 11;20(8):e0328729. doi: 10.1371/journal.pone.0328729 (PMC12338819; doi:10.1371/journal.pone.0328729)
Supplement: Attachment — Submitted filename: Response to Reviewers.pdf [file pone.0328729.s002.pdf]

Response to Reviewers' Comments for Manuscript ID: PONE-D-25-16284

Secure Analysis in Cognitive Satellite-Terrestrial Networks With Untrusted LEO Relaying and Friendly Jamming

Addressed comments for publication in PLOS ONE
by

Peng Zhang and Jin Gao

Dear Prof. Neng Ye and Reviewers,

First of all, we would like to sincerely thank you for your time and effort in handling the review process of our manuscript. In the revised version, we have highlighted all major modifications in blue for your convenience. Based on the valuable suggestions and comments, the primary changes are summarized as follows:

1. We have substantially rewritten the Introduction section to critically analyze recent works on secure terrestrial-satellite communications. We have also clearly pointed out how our work differs from existing studies and highlighted the resulting technical challenges.
2. We have provided a detailed explanation of why the decode-and-forward (DF) relaying strategy, although potentially offering better relay performance, is not applicable under the considered system model. Additionally, we have discussed the feasibility of extending our analytical model to future studies on covert communications.

Please refer to the detailed point-by-point response for further clarification. We once again express our sincere gratitude for the insightful feedback from both the Editor and the Reviewers, which helped us improve the quality of the manuscript.

Yours Sincerely,

Peng Zhang and Jin Gao

Authors' Response to Reviewer 1

Overall Evaluation:
It is highly recommended to enrich the references and consider more recent, 2024-2025, publications on the field. You should study more update papers and compare them with your achievement for the sake of more acceptable presentation.

Response: Thank you very much for your valuable suggestion. We would like to kindly point out that our manuscript already cites and discusses a substantial number of recent and relevant works, including many from 2024–2025. In particular, we have thoroughly reviewed and compared these recent studies with our own contributions in the Introduction section, especially in the parts highlighted in blue in the revised manuscript. We have clearly articulated the distinctions between our work and the existing literature to emphasize the novelty and significance of our contributions. We hope that these clarifications address your concern.

Thank you again for your valuable time invested in improving our submission.

Authors' Response to Reviewer 2

Overall Evaluation:
1. The article aligns well with the journal's scope and is presented in an organized manner, making it easy to read and follow.
2. The authors have investigated physical layer security in a cognitive satellite-terrestrial network where a ground user transmits confidential data to a Geostationary Earth Orbit satellite via an untrusted Low Earth Orbit relay, adopting the Amplify-and-Forward relay strategy.
3. A friendly LEO jammer generates artificial noise to mitigate eavesdropping risks from the relaying LEO satellite. The secrecy rate is assessed under cognitive radio constraints, considering interference thresholds, relay gain, and GU-LEO link shadowing.
4. Numerical results indicate that raising the interference threshold enhances the secrecy rate but levels off once the maximum transmit power constraint at the GU is reached.
5. The authors have concluded that when the maximum power constraint is removed, the interference threshold can be adjusted to optimize the secrecy rate. Additionally, lighter shadowing conditions enhance the secrecy rate, even though they improve the channel conditions for the untrusted relay.
6. The methodology is clearly presented, with extensive explanations and discussions of both the method and the results.
However, I suggest the authors address the following issues:

Response: We sincerely thank the reviewer for the positive evaluation of our work. We have carefully considered all the remaining comments and have provided detailed responses accordingly.

Comment 1:
The authors need to compare the proposed analysis's performance with that of existing literature to demonstrate its robustness.

Response: Thank you very much for your constructive suggestion.

We have substantially revised the Introduction section to better discuss the most recent related works and clearly highlight the differences between our analysis and the existing literature.

As discussed in the Introduction, our considered uplink cognitive radio (CR) satellite-terrestrial scenario is primarily motivated by the recent work [Ref. 7], published in 2024, where the secondary user transmits messages to satellites under CR-based power control.

We would like to clarify that, to the best of our knowledge, very few existing studies have investigated the secrecy performance of this specific uplink CR satellite-terrestrial setup. Therefore, direct performance comparisons with prior literature are limited due to the novelty of the scenario.

We hope this explanation adequately addresses your concern.

Comment 2:
I suggest the authors slightly revise the language of the article. The attached file includes many suggestions.

Response: We sincerely thank you for your careful reading and detailed suggestions on the writing. Following your annotated file, we have carefully revised the manuscript to address all your comments and improve the clarity and quality of the language.

Thank you again for your valuable time invested in improving our submission.

Authors' Response to Reviewer 3

Overall Evaluation:
The manuscript entitled "Secure analysis in cognitive satellite-terrestrial networks with untrusted LEO relaying and friendly jamming" investigates the secrecy rate in cognitive-radio hybrid satellite-terrestrial networks and is well organized with dedicated sections on technological overviews. Here are some issues that the reviewer conserns.

Response: We sincerely thank the reviewer for the positive evaluation of our work. We have carefully considered all the remaining comments and have provided detailed responses accordingly.

Comment 1:
The Introduction spends too much time detailing the general pros and cons of LEO satellites for communication without critically analyzing recent works on secure communications. It fails to highlight the key challenges and advances in the field.

Response: We sincerely appreciate the reviewer's constructive comment.

We have substantially revised the Introduction section, almost rewriting it, to provide a more focused and critical discussion of the latest relevant works on secure terrestrial-satellite communications. In particular, we have emphasized the key challenges and recent advances in the field, especially in the parts highlighted in blue in the revised manuscript.

We hope the revised Introduction now better aligns with the reviewer's expectations.

Comment 2:
In "Numerical Results and Discussion," the manuscript thoroughly examines how different artificial-noise power levels and shadowing conditions affect the secrecy rate, but relies solely on Monte Carlo simulations. It is recommended to include comparisons with data from actual communication scenarios.

Response: Thank you very much for your insightful suggestion.

We would like to clarify that our manuscript follows the standard practice in the field by using Monte Carlo simulations to verify the theoretical expressions and provide numerical comparisons. This approach has also been adopted in the later-cited reference "Cooperative Secure Transmission for Satellite Downlink: A Pas de Deux between Beam Pointings."

The main contribution of our work lies in deriving closed-form expressions for the secrecy rate based on a well-established terrestrial-satellite Rician-shadowed channel model. We then validate the accuracy of the proposed expressions using Monte Carlo simulations.

While we agree that real-world data are valuable, using data from actual communication scenarios introduces uncertainty because the statistical correspondence between the real data and the adopted Rician-shadowed model is unknown. This would prevent a meaningful and direct comparison between the theoretical secrecy rate derived in our work and the empirical secrecy rate computed from such data.

We hope this explanation clarifies our approach and justifies our choice of methodology.

Comment 3:
The Conclusions mention only further parameter optimization under the amplify-and-forward scheme. The authors should also consider whether their closed-form expressions can be applied to alternative security strategies, such as decode-and-forward relaying.

Response: Thanks for your comment.

We would like to clarify that our work specifically considers an untrusted relay scenario. In such a case, applying a decode-and-forward (DF) strategy is not appropriate because the untrusted relay must decode the message from the legitimate user before forwarding it to the destination.

If the untrusted relay successfully decodes the message, the entire transmitted information would be compromised, as it would be fully exposed to the untrusted relay. On the other hand, if the relay fails to decode, the legitimate communication link is disrupted, and the secrecy rate becomes zero.

Therefore, the DF scheme is incompatible with the security assumptions of our system model. For this reason, we focus on the amplify-and-forward (AF) relaying strategy, which is more suitable for untrusted relay scenarios in physical layer security studies. We have also briefly discussed this issue in the revised version, which is also shown below.

Revision in the first paragraph of the system model section

The DF protocol is not considered in this work, as it requires the relay to decode the legitimate user's message before forwarding [17]. In the untrusted relay scenario studied here, this would lead to full exposure of confidential information, making DF unsuitable.

Comment 4:
Regarding the secure-capacity analysis in the manuscript, the authors may expand their research to some recent studies, such as "Achieving Positive Rate of Covert Communications Covered by Randomly Activated Overt Users" derives a tighter upper bound on the total variation distance for randomly activated overt users in both single-frame and multi-frame transmission scenarios, as well as spatially averaged secure capacity in "Cooperative Secure Transmission for Satellite Downlink: A Pas de Deux between Beam Pointings", etc. The authors may refer to these papers to improve their research.

Response: Thank you very much for recommending these valuable references.

We have carefully reviewed the suggested works and have included a discussion of them in the revised manuscript. In particular, please refer to the blue-highlighted parts in the Introduction and the discussion provided in Remark 1, where we comment on their relevance to our secure-capacity analysis and clarify how our work differs and complements these recent studies.

We appreciate your guidance in helping us improve the depth and positioning of our research.

Thank you again for your valuable time invested in improving our submission.
